# Maternal Protein Restriction Alters the Expression of Proteins Related to the Structure and Functioning of the Rat Offspring Epididymis in an Age-Dependent Manner

**DOI:** 10.3389/fcell.2022.816637

**Published:** 2022-04-19

**Authors:** Marilia Martins Cavariani, Talita de Mello Santos, Luiz Gustavo de Almeida Chuffa, Patrícia Fernanda Felipe Pinheiro, Wellerson Rodrigo Scarano, Raquel Fantin Domeniconi

**Affiliations:** Department of Structural and Functional Biology, Institute of Biosciences of Botucatu, São Paulo State University (UNESP), São Paulo, Brazil

**Keywords:** maternal protein restriction, epididymis, hormone receptors, src, CLDN-1, postnatal development

## Abstract

Nutrition is an environmental factor able to activate physiological interactions between fetus and mother. Maternal protein restriction is able to alter sperm parameters associated with epididymal functions. Since correct development and functioning of the epididymides are fundamental for mammalian reproductive success, this study investigated the effects of maternal protein restriction on epididymal morphology and morphometry in rat offspring as well as on the expression of Src, Cldn-1, AR, ER, aromatase p450, and 5α-reductase in different stages of postnatal epididymal development. For this purpose, pregnant females were allocated to normal-protein (NP—17% protein) and low-protein (LP—6% protein) groups that received specific diets during gestation and lactation. After weaning, male offspring was provided only normal-protein diet until the ages of 21, 44, and 120 days, when they were euthanized and their epididymides collected. Maternal protein restriction decreased genital organs weight as well as crown-rump length and anogenital distance at all ages. Although the low-protein diet did not change the integrity of the epididymal epithelium, we observed decreases in tubular diameter, epithelial height and luminal diameter of the epididymal duct in 21-day-old LP animals. The maternal low-protein diet changed AR, ERα, ERβ, Src 416, and Src 527 expression in offspring epididymides in an age-dependent manner. Finally, maternal protein restriction increased Cldn-1 expression throughout the epididymides at all analyzed ages. Although some of these changes did not remain until adulthood, the insufficient supply of proteins in early life altered the structure and functioning of the epididymis in important periods of postnatal development.

## 1 Introduction

One of the first concepts to emerge in the literature relating the conditions of the intrauterine environment to changes in progeny development was the “fetal origin of diseases” theory (Barker, 1995a). The “fetal origin” hypothesis proposes that changes in fetal nutrition and hormonal status result in adaptations during development capable of altering the structure, physiology and metabolism of the embryo, thus predisposing it to cardiovascular, metabolic and endocrine diseases in adult life (Barker, 1995b; 2007). Currently, the concept that intrauterine environmental conditions are able to influence the establishment of adulthood diseases is known as the Developmental Origins of Health and Disease (DOHaD) (Jazwiec and Sloboda, 2019).

It is well established that maternal nutrition has a significant impact on development and fetal health, since during gestation and lactation, the fetus depends exclusively on the mother to supply its nutritional requirements (McArdle et al., 2006; Jazwiec and Sloboda, 2019; Lindsay et al., 2019). Therefore, nutrition during pregnancy is an environmental factor able to activate physiological interactions between the fetus and mother that are often mediated through hormonal signaling and may cause epigenetic alterations in genes that regulate the target tissues of these hormones. These interactions may modify fetal growth and metabolic character, establishing the basis for several diseases when there is inconsistency between gestational and postnatal nutrient availability (Fleming et al., 2015).

Hormonal signaling changes during sensitive periods of development may alter the development of specific fetal tissues, lead to long-lasting changes in tissue sensitivity to hormones or even change hormone secretion (Godfrey and Barker, 2000; Console et al., 2001; Peixoto-Silva et al., 2011; Rinaldi et al., 2018). Rats subjected to maternal protein restriction during intrauterine development show changes in testosterone, estradiol and aldosterone concentration that result in significant impacts on organs and functions of the genital system (Zambrano et al., 2005; Colombelli et al., 2017; Santos et al., 2018; Cavariani et al., 2019).

The epididymis is an androgen-dependent organ anatomically divided into the initial segment, caput, corpus, and cauda. Each epididymal region is responsible for characteristic functions such as secretion, endocytosis, absorption and acidification, which lead to the establishment of a specific intraluminal environment suitable for the concentration and maturation of the spermatozoa produced by the testes (Cosentino and Cockett, 1986; Hermo and Robaire, 2002; Gatti et al., 2004; Robaire and Hinton, 2015).

The epididymis originates from the proximal part of the Wolffian duct, acquiring a coiled appearance by the influence of testosterone (Moore et al., 2016). The enzyme 5α-reductase mediates the conversion of testosterone to dihydrotestosterone (DHT), which is the most active regulator of epididymal cell function being five to ten times more potent than testosterone with a higher affinity to androgen receptor (AR) (Gloyna and Wilson, 1969; Cohen et al., 1981; Rhoden et al., 2002; Hackel et al., 2005; Traish et al., 2015). Both testosterone and DHT bind to the AR being responsible for activating different processes (Silverthorn, 2016).

Estrogen is also actively involved in the development and functions of the epididymis (Hess and Zhou, 2002; Cooke et al., 2017; Hess et al., 2021). In immature males, the main source of estrogen is the Sertoli cells, while in adults, germ cells show high expression of the enzyme aromatase cytochrome p450, which converts androgen into estrogen (Nitta et al., 1993; Hess et al., 1995; Janulis et al., 1998; Ghosh et al., 2009; Russell and Grossmann, 2019). Estrogens mediate their effects by binding to the nuclear receptors termed ERα and ERβ in the epididymis (Kuiper et al., 1996; Pelletier et al., 2000; Zaya et al., 2012).

A maternal low-protein diet during gestation and lactation is able to induce testicular, prostatic and sperm changes in adult animals (Zambrano et al., 2005; Toledo et al., 2011; Rodriguez-Gonzalez et al., 2012; Rodriguez-Gonzalez et al., 2014; Colombelli et al., 2017; Santos et al., 2018). Regarding sperm alterations, it has been observed that maternal protein restriction caused alterations mainly associated with epididymal functions such as sperm motility, viability and concentration (Toledo et al., 2011; Rodriguez-Gonzalez et al., 2014). However, although studies have shown effects of protein restriction associated with epididymal functions, the causes of these alterations have not yet been entirely clarified.

During gestation, an increase in protein turnover occurs to enable rapid embryo growth; therefore, adequate intake of protein at this stage is recommended (Jolly et al., 2004). Although some effects of protein restriction are mediated by hormonal effects, several others are direct consequences of changes in substrate availability.

Srcs are nonreceptor protein kinases that act in multiple cellular environments, playing key roles in the regulation of signal transduction through several cell surface receptors (Parsons and Parsons, 2004). In the epididymis, Srcs stand out as regulators of epididymal development in addition to playing important roles in spermatozoa changes that occur during epididymal transit and sperm maturation within the organ (Krapf et al., 2012; Xu et al., 2016).

The structure and integrity of the blood–epididymal barrier are essential for the maintenance of epididymal intraluminal environment specificity (Hoffer and Hinton, 1984; Dufresne and Cyr, 2007). In this context, the claudins (Cldns) are a family of transmembrane proteins that are essential components of the tight junctions that compose this barrier (Gregory and Cyr, 2006; Cyr et al., 2007). Although several claudins integrate tight junctions, studies have demonstrated the presence of Cldn-1 in all regions of the rat epididymis. In addition, Cldn-1 appears throughout all interfaces of adjacent epithelial cells and throughout all basal plasma membrane extensions, suggesting that Cldn-1 plays a role as an adhesion molecule (Gregory et al., 2001; Dufresne and Cyr, 2007; Kim and Breton, 2016). Cldn-1 knockout mice die of dehydration soon after birth due to lack of the epidermal barrier, thus demonstrating that Cldn-1 is indispensable for survival and cannot be replaced by any of the other tight junction proteins (Furuse et al., 2002).

Insufficient protein intake in a large proportion of the human population due to cultural or economic reasons is a global concern. For this reason, the model of protein restriction remains one of the most characterized early growth restriction models studied until now (Fleming et al., 2015; Semba, 2016; Herring et al., 2018). Maternal protein restriction is able to alter sperm parameters associated with epididymal function, such as sperm motility, viability and concentration. Since the correct development and functioning of the epididymis are fundamental for mammalian reproductive success, this study investigated the effects of maternal protein restriction on epididymal morphology and morphometry in rat offspring as well as on the expression of Src, Cldn-1, AR, ERα, ERβ, aromatase p450, and 5α-reductase in different stages of postnatal epididymal development.

Considering the direct effects of maternal protein restriction related to changes in substrate availability and hormonal signaling, we hypothesized that the sperm alterations associated with the correct functioning of epididymis previously observed in this experimental model are related to differential expression of membrane proteins and hormone receptors essential for the epididymal performance in specific periods of postnatal development.

## 2 Materials and Methods

### 2.1 Animals and Experimental Design

Wistar rats 45 days in age (male *n* = 20; female *n* = 38) were obtained from the Central Biotherium, Institute of Biosciences/Campus of Botucatu, UNESP–São Paulo State University. The animals were housed in polyethylene cages (43 × 30 × 15 cm) lined with an autoclaved pine sawdust substrate under controlled conditions of temperature and light (12-h light/dark cycle). The animals were maintained with free access to water and commercial solid food for rodents.

For mating, two sexually receptive females and one breeder male rat at 95 days of age were kept in maternity boxes overnight. The next morning, pregnancy was confirmed by the presence of spermatozoa in vaginal smears; this day was designated gestational day (GD) 0. Pregnant females were housed individually in standard rat cages and divided into a low-protein (LP) group (*n* = 19) and a normal-protein (NP) group (*n* = 19). The LP mothers were fed a low-protein diet (6% protein), while the NP mothers were fed a normal-protein diet (17% protein), both provided from Pragsoluções Biociências (Jaú, SP, Brazil). The NP and LP diets were prepared following the recommendation by the American Institute of Nutrition (AIN 93-G) (Reeves et al., 1993). The diets are isocaloric and widely used for the study of maternal protein restriction (Rinaldi et al., 2013; Colombelli et al., 2017; Santos et al., 2018; Cavariani et al., 2019). Both groups received their respective diets ad libitum (Table 1).

**TABLE 1:** Composition of diets offered to animals during gestation and lactation.

Components *	Normoprotein diet (17%)	Low-protein diet (6%)
Casein (84% of protein)**	202.00	71.50
Cornstarch	397.00	480.00
Dextrin	130.50	159.00
Sucrose	100.00	121.00
Soy oil	70.00	70.00
Fiber (microcellulose)	50.00	50.00
Mineral Blend ***	35.00	35.00
Vitamin Blend ***	10.00	10.00
L-cystine	3.00	1.00
Choline chloride	2.50	2.50

* Diet for the gestation phase in rodents - AIN-93G.

** Corrected values according to protein content in casein.

*** According to AIN-93G.

**FIGURE 1:**
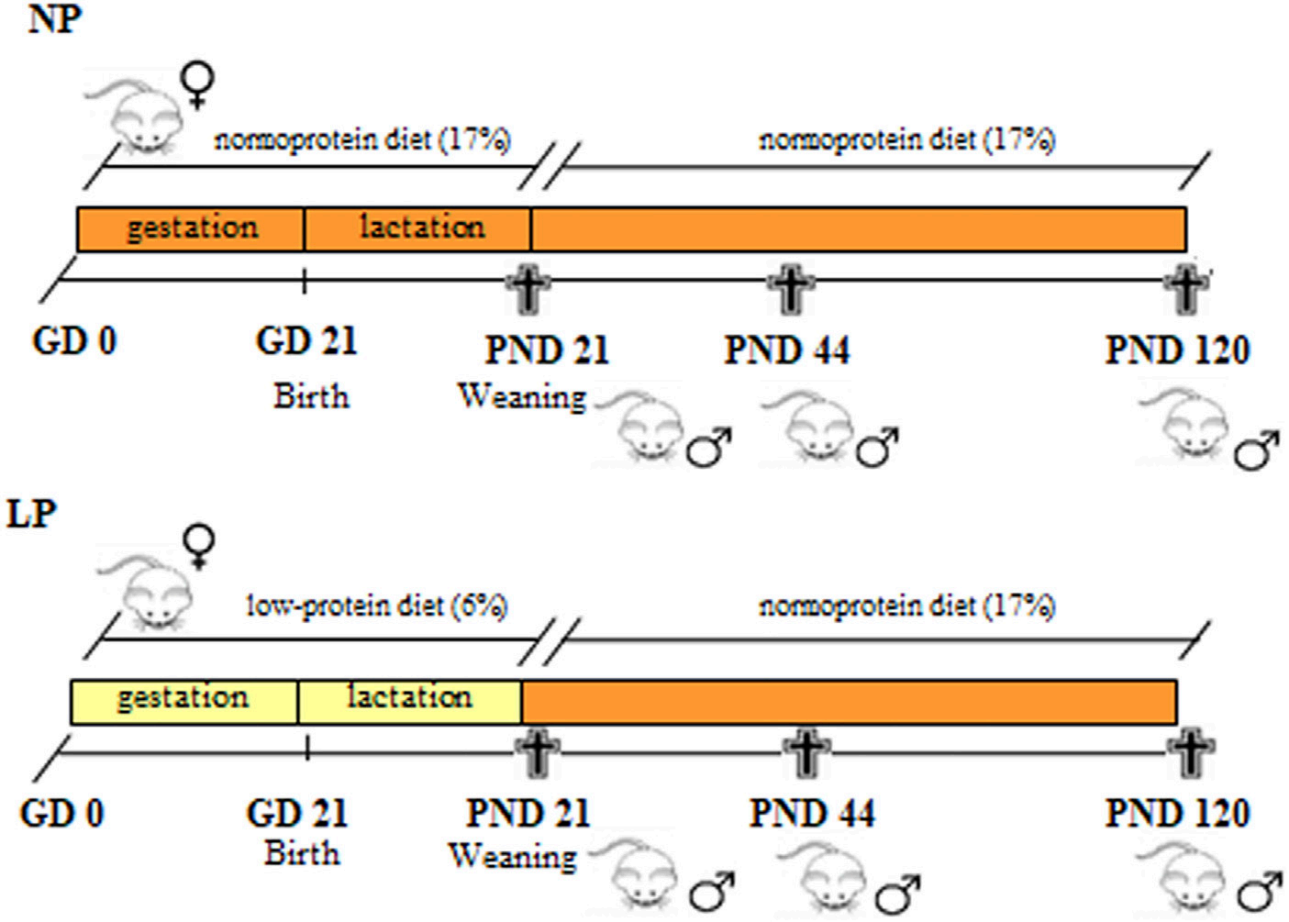
Experimental design. Pregnant rats received a low-protein diet (LP group) or a normal-protein diet (NP group) ad libitum from the GD 0 until PND 21 (during gestation and lactation). After weaning, male pups from both groups received a standard diet until PNDs 21, 44 and 120.

The normal-protein and low-protein diets were offered to the indicated groups during gestation and lactation, from GD 0 until the offspring were weaned at postnatal day 21. To ensure equal availability of nourishment, only eight pups per litter (preferably males) were maintained with each mother. After weaning, the LP and NP male offspring received the standard diet for rodents until the age of 21 (NP, *n* = 17; LP, *n* = 22), 44 (NP, *n* = 12; LP, *n* = 10) and 120 (NP, *n* = 13; LP, *n* = 12) days, when they were decapitated and their blood and epididymides collected (Figure 1). Each individual was chosen from different litters to ensure a representative sampling. The ages of the animals at euthanasia were chosen based on three different phases of the epididymis postnatal development: at 21 days of age, peak epididymal cell differentiation occurs; at 44 days of age, the final period of epididymal differentiation and the beginning of epididymal expansion occurs; and at 120 days of age, the epididymides are well differentiated, and the animals are considered sexually mature (Picut et al., 2018).

The experimental procedures were approved by the Ethics Committee on Animal Experimentation (EAEC) of the Institute of Biosciences of Botucatu (number 797-CEUA) in addition to being in accordance with the Ethical Principles on Animal Experimentation adopted by the Brazilian College of Animal Experimentation (COBEA).

### 2.2 Gestational Performance

Gestational performance is an important parameter in studies that use maternal malnutrition as an experimental model. This parameter is assessed on the day of offspring birth (PND 1) and presents relevant information regarding gestation and offspring development.

In PND 1, male pups from each litter were separated, weighed individually and had the anogenital distance (AGD) and crown-rump length (CRL) recorded using a digital caliper (Western^®^). Relative AGD was obtained by dividing the absolute AGD by the CRL. For each litter of NP and LP groups, the number of male and female pups were counted in addition to the total number of pups (data not shown).

### 2.3 Hormonal Assay

Through narcosis induced in a CO_2_ chamber, the animals were anesthetized and then decapitated (for cervical vessel rupture), and the blood was collected. Blood serum was obtained by centrifugation at 14,000 rpm for 20 min at 4°C. Serum samples were assayed for estradiol levels using chemiluminescence with a specific kit provided by Beckman Coulter, Inc. (REF B84493, Brea, CA, United States). The lower and higher limits of detection for estradiol were 0.017 ng/dl and 6.9 ng/dl, respectively. All samples were assayed at the same time to prevent interassay variation.

### 2.4 Morphological and Morphometrical Analyses of the Epididymis

Following euthanasia, the right epididymides were individually collected, immediately fixed in 10% buffered formalin (0.1 M phosphate buffer, pH 7.3) for 24 h and then washed in running water for 24 h. Then, the epididymides were dehydrated in a graded series of ethanol solutions, diaphanized in N-butyl alcohol and embedded in Paraplast (Paraplast Plus, St. Louis, MO, United States). Epididymal sections 5 μm thick were made using a LEICA RM 2165 μm (Leica Biosystems, Nußloch, Germany). Four blocks were cut for the NP and LP groups at each age; the blocks from the animals at PND 21 were cut into serial sections, while the blocks from the animals at PNDs 44 and 120 were cut into semiserial sections.

For all ages (21, 44 and 120 days), four slides from each of the four animals of each group (LP and NP) were stained with hematoxylin and eosin (H&E). The slides were scanned with a 3DHistech Pannoramic MIDI, analyzed for epithelial and interstitial integrity and then photographed using the Pannoramic Viewer program.

Morphometric analyses were conducted by adapting the procedures described by Serre and Robaire (1998). For this technique, the same slides used for analysis of epididymal morphology were analyzed. Briefly, the epithelial height, luminal diameter and tubular epididymal diameter was measured in at least 10 transverse sections of epididymal tubules in each of the epididymal regions using the Pannoramic Viewer program.

### 2.5 Immunohistochemistry

For immunohistochemistry, sections of epididymides from LP and NP animals (n = 4 animals/group at each age) were deparaffinized (40 min in the oven at 60 °C), incubated in xylene and hydrated with decreasing concentrations of ethanol. Antigen retrieval was performed in Tris-EDTA buffer (pH 9.0) in a water bath for AR and ERα at PNDs 21 and 44 and in 0.01 M sodium citrate buffer (pH 6.0) in a pressure cooker for AR and ERα at PND 120 and for ERβ at all ages. After endogenous peroxidase was blocked (H2O2, 0.3% in methanol), the tissues were incubated with 3% BSA for 1 h. The sections of epididymis were incubated overnight at 4 °C with 1:100 dilutions of the following primary antibodies in 1% BSA: anti-AR (Millipore, Temecula, CA, United States), anti-ERα (Millipore, Temecula, CA, United States) and anti-ERβ (Millipore, Temecula, CA, United States). Early the next morning, the sections were washed with PBS buffer and then incubated with 1:200 dilutions of a peroxidase-conjugated anti-Rb secondary antibody (Sigma, St. Louis, MO, United States) for AR and ERα at PNDs 21 and 44 and a biotinylated anti-Rb secondary antibody (Sigma, St. Louis, MO, United States) for AR and ERα at PND 120 and for ERβ at all ages in 1% BSA for 2 h. The sections labeled with biotinylated antibodies were incubated for 45 min with an ABC complex (ABC Vectastain^®^ kit, Burlingame, CA, United States) and subsequently washed with PBS. The immunoreactive components were reacted with diaminobenzidine (DAB; Sigma, St. Louis, MO, United States), and counterstaining was performed with hematoxylin. Finally, a 3D Histech Pannoramic MIDI was used to scan the slides, which were photographed and analyzed using the Pannoramic Viewer program. Negative controls were obtained from each reaction using 1% BSA and omitting the primary antibody in the overnight incubation step.

### 2.6 Western Blot Analysis

The left epididymides from NP and LP animals at the ages of 21, 44, and 120 days (*n* = 5) were divided into the initial segment plus caput (SI + CP) and the corpus plus cauda (CO + CD). The samples were homogenized with RIPA lysis buffer (Bio-Rad, Hercules, CA, United States) supplemented with a protease inhibitor cocktail (Sigma, St. Louis, MO, United States). Subsequently, the homogenate was centrifuged at 14,000 rpm for 20 min to remove the cell debris, and the supernatant was then collected. Total protein concentrations were measured using the Bradford colorimetric method (Bradford, 1976). Afterwards, 70 µg of protein was added to 1.5X Laemmli buffer, and the individual proteins were then separated by 4–15% polyacrylamide gel electrophoresis (SDS-PAGE) for 90 min at 120 V. Following electrophoresis, the proteins were electrotransferred to nitrocellulose membranes in a wet system at 350 mA. The membranes were blocked with TBS-T solution containing 3% milk (Molico^®^) for 1 h at room temperature. The membranes were incubated overnight at 4°C with the following primary antibodies diluted in TBS-T: anti-AR (1:1,000 dilution; Millipore, Temecula, CA, United States), anti-ERα (1:200 dilution; Millipore, Temecula, CA, United States), anti-ERβ (1:300 dilution; Millipore, Temecula, CA, United States), anti-Src 416 (1:500 dilution; Cell Signaling, Danvers, MA, United States), anti-Src 527 (1:1,000 dilution; Cell Signaling, Danvers, MA, United States), anti-Cldn-1 (1:1,000 dilution; Thermo Fisher Scientific, Rockford, IL, United States) and anti-β-actin (1:800 dilution; Santa Cruz, Santa Cruz, CA, United States). Early the next morning, the membranes were washed with TBS-T solution and then incubated with a 1:2000 dilution of an anti-Rb secondary antibody for AR, ERα, ERβ and Cldn-1 (Sigma, St. Louis, MO, United States), a 1:5,000 dilution of an anti-Rb secondary antibody for Src 416 and Src 527 (Sigma, St. Louis, MO, United States) and an anti-goat secondary antibody (1:6,000 dilution; Sigma, St. Louis, MO, United States) diluted in TBS-T for 2 h before being washed with TBS-T solution. Subsequently, the immunoreactive bands were developed using a chemiluminescence kit (Amersham ECL^™^ Western Blotting Detection Reagent Select) from GE Healthcare^®^ and analyzed semiquantitatively by optical densitometry with ImageJ analysis software for Windows. The values obtained for each band of AR, ERα, ERβ, Src 416, Src 527, and Cldn-1 were normalized to the β-actin density, and the data are presented as the mean ± S.E.M. The immunoblotting data are presented as optical densitometry index (% band intensity).

### 2.7 Statistical Analysis

GraphPad Prism^®^ software (version 5.00, Graph Pad, Inc., San Diego, CA) was used to perform the statistical analyses. At all analyzed ages, comparisons between the LP and NP groups were performed using the Mann-Whitney test for nonparametric data and Student’s t-test for parametric data. All data are presented as the mean ± S.E.M., and statistical significance was set at *p* < 0.05.

## 3 Results

### 3.1 Maternal Low-Protein Diet Promotes Changes in Gestational Performance as Well as Genital Organ Weight in Male Offspring

Maternal protein restriction during gestation and lactation did not alter the number of male pups but significantly decreased the body weight of the pups at birth (0.91-fold decrease in the LP group compared with the NP group, these data were presented as Supplementary Materials in an article previously published by our research group (Cavariani et al., 2019) (Table 2).

**TABLE 2:** Body weight, crown-rump length and absolute and relative anogenital distance of males at birth (PND 1). NP, *n* = 17 litters/group. LP, *n* = 19 l/group. Values expressed as means ± S.E.M. *p < 0.05. Student t-teste was used to asses significance of differences in parametric data (a) and Mann-whitney was used to asses significance of differences in non-parametric data (b).

*Parameters*	*NP*	*LP*
Number of male pups	5.29 ± 0.43	5.47 ± 0.52^a^
Body weight (g)	6.33 ± 0.12	5.79 ± 0.10^a^*
Anogenital distance (mm)	2.99 ± 0.03	2.59 ± 0.05^b^*
Crown-rump length (mm)	49.13 ± 0.32	47.46 ± 0.21^a^*
Relative anogenital distance (mm)	0.061 ± 0.001	0.055 ± 0.001^b^*

With regard to the other parameters analyzed on the day of offspring birth, this experimental model caused a significant reduction in crown-rump length (CRL) (0.97-fold reduction in the LP group compared with the NP group) and absolute anogenital distance (AGD) (0.87-fold reduction in the LP group compared with the NP group), as well as in relative AGD (0.9-fold reduction in the LP group compared with the NP group) (Table 2).

**FIGURE 2:**
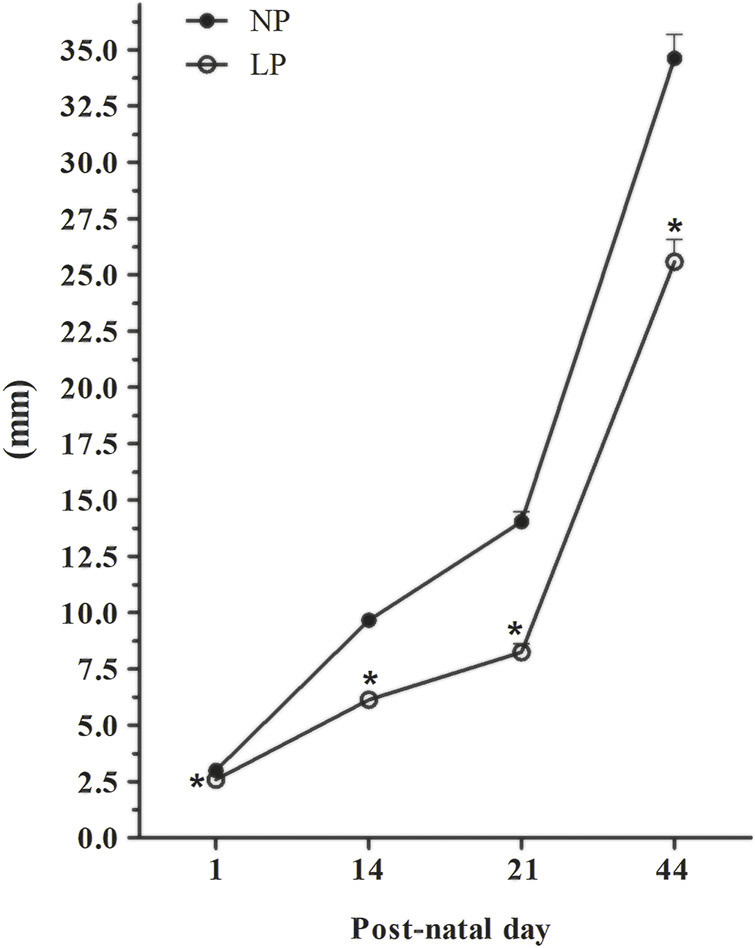
Evolution of the anogenital distance. PND 1: NP, *n* = 17, LP, *n* = 19; PND 14: NP, *n* = 19, LP, *n* = 26; PND 21: NP, *n* = 17, LP, *n* = 22; PND 44: NP, *n* = 12, LP, *n* = 10. Evolution of AGD in animals whose mothers received normal-protein and low-protein diets during gestation and lactation. The values are expressed as the mean ± S.E.M. *p < 0.05. Student t-test was used to assess the significance of parametric data, and Mann-Whitney test was used to assess the significance in nonparametric data.

At PNDs 21 and 44, AGD remained significantly reduced in LP animals compared with NP animals (PND 21: 0.59-fold reduction in the LP group compared with the NP group; PND 44: 0.74-fold reduction in the LP group compared with the NP group) (Figure 2).

Although CRL remained significantly reduced in LP animals at PNDs 21 and 44 (PND 21: 0.67-fold reduction in the LP group compared with the NP group; PND 44: 0.8-fold reduction in the LP group compared with the NP group) (Supplementary Table 1), there was no significant difference in relative AGD between the groups at both ages (PND 21: 0.9-fold reduction in the LP group compared with the NP group; PND 44: 0.9-fold reduction in the LP group compared with the NP group) (Supplementary Table 1).

Regarding genital organ weight, the low-protein diet caused a significant decrease in the absolute weights of the testes and epididymides at all analyzed ages (testis weight: 0.41-fold decrease in the LP group compared with the NP group at PND 21; 0.55-fold decrease in the LP group compared with the NP group at PND 44; 0.79-fold decrease in the LP group compared with the NP group at PND 120. Epididymis weight: 0.52-fold decrease in the LP group compared with the NP group at PND 21; 0.51-fold decrease in the LP group compared with the NP group at PND 44; 0.84-fold decrease in the LP group compared with the NP group at PND 120) (Table 3).

**TABLE 3:** Body weight and absolute and relative genital organs weight at PND day 21, 44 and 120. PND 21: NP, n = 17, LP, n = 22; PND 44: NP, n = 12, LP, n = 10; PND 120: NP, n = 10, LP, n = 10. The values are expressed as the mean ± S.E.M. *p < 0.05. Student t-tests was used to analyze the significance of differences in parametric data (^a^), and Mann-Whitney tests was used to analyze the significance of differences in nonparametric data (^b^).

*Parameters*	PND 21		PND 44		PND 120	
	NP (*n* = 17)	LP (*n* = 22)	NP (*n* = 12)	LP (*n* = 10)	NP (*n* = 10)	LP (*n* = 10)
Body weight (g)	58.63 ± 1.59	19.29 ± 0.74^b^*	219.30 ± 4.99	122.50 ± 4.84^b^*	492.00 ± 13.96	399.90 ± 7.33^a^*
Testes (mg)	110.40 ± 2.79	45.30 ± 2.39 ^b^*	944.10 ± 35.77	516.10 ± 27.03^a^*	1802 ± 68.05	1415 ± 39.23^a^*
Testes (mg/100 g)	189.50 ± 5.00	235.2 ± 8.80^a^*	412.30 ± 21.29	420.20 ± 12.65^b^	375.80 ± 14.87	355.30 ± 12.94^a^
Epididymis (mg)	17.29 ± 0.82	8.93 ± 0.40^b^*	141.70 ± 8.58	72.52 ± 4.27^a^*	826.40 ± 34.26	697.40 ± 21.20^a^*
Epididymis (mg/100 g)	28.65 ± 0.82	46.50 ± 1.62^b^*	61.98 ± 2.06	58.87 ± 1.67^a^	172.40 ± 7.87	175.40 ± 2.94^a^
Ventral prostate (mg)	21.49 ± 1.57	8.08 ± 0.59^b^*	103.70 ± 11.36	44.40 ± 5.70^a^*	928.50 ± 79.31	769.80 ± 63.45^a^
Ventral prostate (mg/100 g)	39.34 ± 3.42	41.67 ± 2.44^a^	46.85 ± 4.45	36.32 ± 4.42^a^	189.10 ± 12.97	190.70 ± 13.48^a^
Seminal vesicle (empty) (mg)	11.28 ± 0.38	4.70 ± 0.29^b^*	100.90 ± 10.94	39.31 ± 3.95^b^*	1472 ± 104.00	1647 ± 104.80^a^
Seminal vesicle (empty) (mg/100 g)	18.77 ± 0.88	24.46 ± 1.30^a^*	45.43 ± 4.34	31.80 ± 2.69^a^*	302.40 ± 27.27	424.40 ± 22.97^a^*

Ventral prostate and empty seminal vesicle absolute weights were significantly reduced in LP animals at PNDs 21 and 44 (ventral prostate weight: 0.38-fold decrease in the LP group compared with the NP group at PND 21; 0.43-fold decrease in the LP group compared with the NP group at PND 44. Empty seminal vesicle weight: 0.42-fold decrease in the LP group compared with the NP group at PND 21; 0.40-fold decrease in the LP group compared with the NP group at PND 44) (Table 3), but there was no significant difference at PND 120 (0.83-fold decrease in ventral prostate weight in the LP group compared with the NP group; 1.12-fold increase in empty seminal vesicle weight in the LP group compared with the NP group) (Table 3).

The relative weights of genital organs were significantly elevated in LP animals at PND 21 (1.24-fold increase in relative testis weight in the LP group compared with the NP group; 1.62-fold increase in relative epididymis weight in the LP group compared with the NP group; 1.06-fold increase in relative ventral prostate weight in the LP group compared with the NP group; 1.30-fold increase in relative empty seminal vesicle weight in the LP group compared with the NP group) (Table 3).

At PND 44, the relative testis weight was slightly elevated in LP animals (1.02-fold increase in the LP group compared with the NP group) (Table 3), while for the epididymis and ventral prostate, the relative weights were slightly reduced in LP animals (0.95-fold decrease in relative epididymis weight in the LP group compared with the NP group; 0.78-fold decrease in relative ventral prostate weight in the LP group compared with the NP group) (Table 3). The relative empty seminal vesicle weight was significantly reduced in LP animals (0.70-fold decrease in the LP group compared with the NP group) (Table 3).

Finally, at PND 120, the relative weights of the epididymis and ventral prostate showed practically no differences between NP and LP animals (Table 3). The relative testis weight was slightly reduced in LP animals (0.95-fold decrease in the LP group compared with the NP group) (Table 3), and the relative empty seminal vesicle weight was significantly elevated in animals whose mothers received a low-protein diet (1.40-fold increase in the LP group compared with the NP group) (Table 3).

### 3.2 Maternal Protein Restriction did Not Change the Integrity of the Epididymal Epithelium or the Organ Interstitium but Changed the Tubular Diameter, Epithelial Height and Luminal Diameter of the Epididymal Duct

**FIGURE 3:**
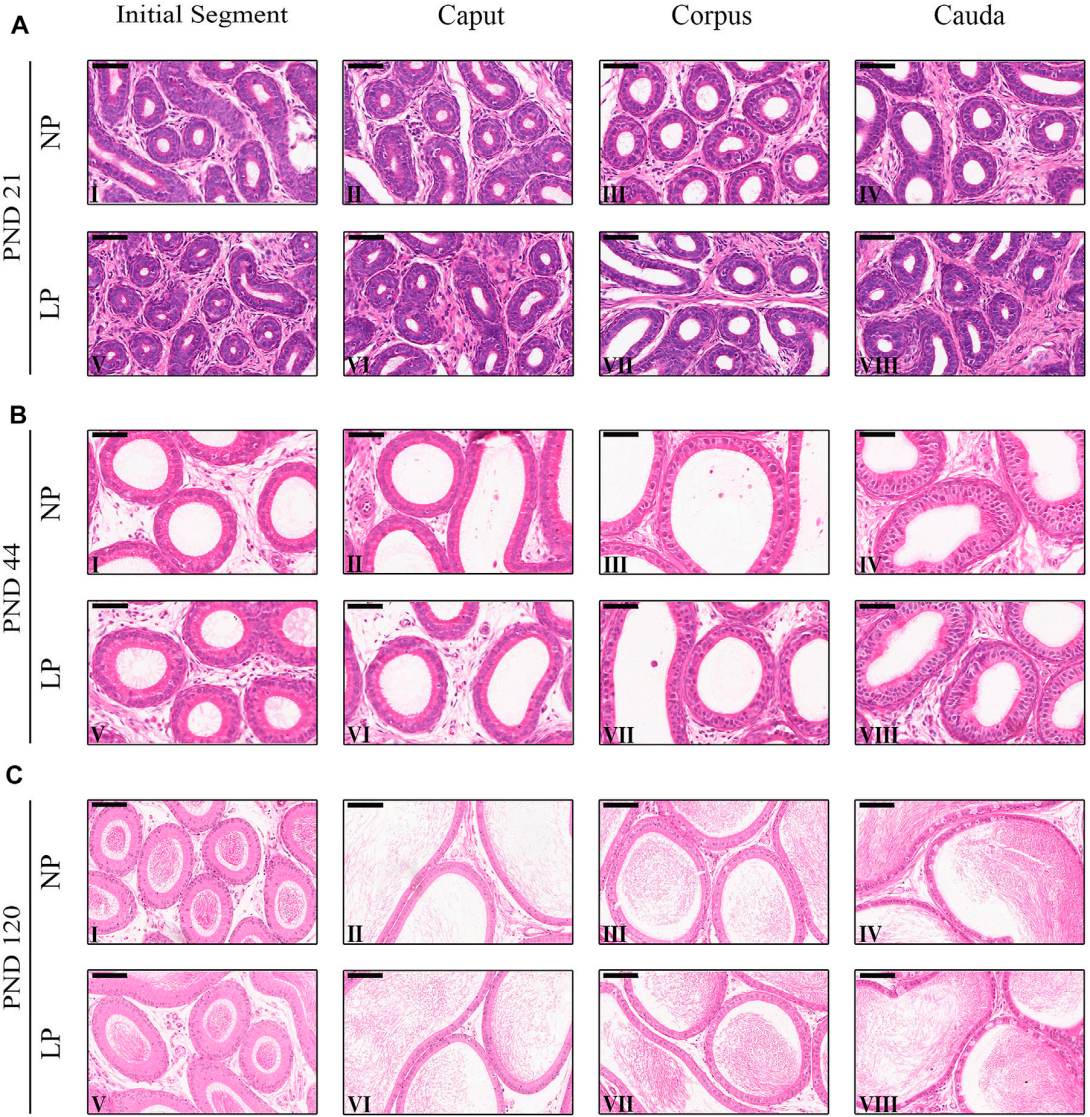
Morphology of the epididymis. **(A)** Staining of the epididymides of NP and LP animals at PND 21 showing the initial segment **(I and V)**, caput **(III and VI)**, corpus **(III and VII)** and cauda **(IV and VIII)**. H&E, Bar = 50 µm. **(B)** Staining of the epididymides of NP and LP animals at PND 44 showing the initial segment **(I and V)**, caput **(III and VI)**, corpus **(III and VII)** and cauda **(IV and VIII)** . H&E, Bar = 50 µm. **(C)** Staining of the epididymides of NP and LP animals at PND 120 showing the initial segment **(I and V)**, caput **(III and VI)**, corpus **(III and VII)** and cauda **(IV and VIII)**. H&E, Bar = 100 µm.

Both the NP and LP groups at PNDs 44 and 120 presented initial segment regions with small tubular diameters and organized epithelia containing principal cells, basal cells, narrow cells, and few apical cells (Figures 3BI,V,CI,V. The epididymal caput also had a well-organized epithelium with the presence of principal cells, basal cells and clear cells. In this region, the tubular diameter was slightly greater than that in the initial segment region (Figures 3BII,VI,CII,VI). With age advancement, the epididymal corpus showed principal cells with slightly more cubic shapes than the principal cells of the initial segment and caput regions in addition to presenting clear cells and basal cells (Figures 3BIII,VII,CIII,VII). In the cauda region, the epithelial height did not differ greatly from the epithelial height of the caput region between the groups of animals at 21 and 44 days but was much reduced in the animals at 120 days. On PNDs 44 and 120, principal cells, basal cells and a greater number of clear cells were observed in the caudal region compared to the other epididymal regions (Figures 3BIV,VIII,CIV,VIII).

LP animals at PND 21 presented reductions in tubular diameter, epithelial height and luminal diameter in all epididymal regions. However, the decrease in tubular diameter in the cauda and the decreases in epithelial height in the initial segment and cauda were not significant at this age (Table 4).

**TABLE 4:** **–** Morphometry of the epididymis. Tubular diameter, epithelium height and luminal diameter at PND 21, 44 and 120. NP, *n* = 4; LP, *n* = 4. Values expressed as means ± S.E.M. *p < 0.05. Student t-teste was used for parametric data (a), and Mann-whitney test was used for non-parametric data (b).

Parameters (µm)								
PND 21 (n = 4)	Initial Segment		Caput		Corpus		Cauda	
	NP	LP	NP	LP	NP	LP	NP	LP
Tubular diameter	40.88 ± 1.59	30.61 ± 0.45^a^*	48.34 ± 2.86	36.64 ± 0.85^a^*	60.60 ± 2.33	44.66 ± 1.45^a^*	58.57 ± 4.86	48.96 ± 4.15^a^
Epithelium height	12.22 ± 0.05	9.73 ± 0.19^b^	13.12 ± 0.78	10.93 ± 0.32^a^*	16.18 ± 0.63	12.27 ± 0.24^a^*	13.57 ± 1.09	14.15 ± 1.47^a^
Luminal diameter	17.68 ± 1.08	10.65 ± 0.29^a^*	21.99 ± 2.32	13.72 ± 0.12^b^*	26.81 ± 1.56	18.36 ± 1.54^a^*	34.45 ± 4.10	20.59 ± 2.34^a^*
***PND 44*** (***n = 4***)	**Initial Segment**		**Caput**		**Corpus**		**Cauda**	
	***NP***	***LP***	***NP***	***LP***	***NP***	***LP***	***NP***	***LP***
Tubular diameter	100.8 ± 4.36	97.91 ± 0.78^a^	131.3 ± 12.69	114.1 ± 7.63^a^	188.1 ± 14.56	147.0 ± 12.50^a^	118.5 ± 2.49	113.1 ± 3.82^a^
Epithelium height	19.44 ± 0.33	19.96 ± 0.47^a^	15.67 ± 0.67	15.86 ± 0.68^a^	17.91 ± 1.42	20.01 ± 1.50^a^	24.63 ± 0.59	22.78 ± 0.25*^a^
Luminal diameter	61.54 ± 4.63	58.51 ± 0.99^a^	99.11 ± 14.26	84.58 ± 7.41^a^	152.6 ± 17.42	108.1 ± 16.98^a^	64.97 ± 1.46	63.94 ± 3.38^a^
***PND 120*** (**n *= 4***)	**Initial Segment**		**Caput**		**Corpus**		**Cauda**	
	***NP***	***LP***	***NP***	***LP***	***NP***	***LP***	***NP***	***LP***
Tubular diameter	172.8 ± 11.26	173.9 ± 7.68^a^	320.5 ± 23.68	318.9 ± 8.31^a^	326.1 ± 8.95	298.4 ± 11.37^a^	289,7 ± 6,12	312,3 ± 19,11^b^
Epithelium height	29.54 ± 0.40	33.38 ± 3.72^a^	22,07 ± 1,31	21,63 ± 0,01^b^	23.02 ± 1.12	23.95 ± 1.15^a^	19.13 ± 1.23	18.85 ± 1.53^a^
Luminal diameter	107.5 ± 5.52	99.77 ± 6.55^a^	276.6 ± 21.32	273.3 ± 9.77^a^	280.2 ± 10.65	252.2 ± 10.54^a^	239.8 ± 10.86	274.4 ± 21.86^a^

At PND 44, the tubular diameter and luminal diameter were decreased nonsignificantly in all epididymal regions of animals whose mothers received a low-protein diet during gestation and lactation. Conversely, the LP animals presented a slight increase in epithelial height in the initial segment, caput and corpus, while in the cauda region, a significant decrease in epithelial height was observed (Table 4).

In 120-day-old animals, maternal protein restriction had slightly decreased the tubular diameter in the caput and corpus regions and slightly increased this parameter in the initial segment and cauda regions. Conversely, epithelial height was increased nonsignificantly in the caput and corpus regions but decreased nonsignificantly in the initial segment and cauda region. Finally, at this age, the luminal diameter was decreased nonsignificantly in all epididymal regions of LP animals, except for the cauda region, in which a slight increase in luminal diameter was observed (Table 4).

### 3.3 Maternal Protein Restriction did Not Alter Estradiol Serum Levels in Male Offspring

In 21-day-old animals, only a slight increase in circulating estradiol was observed in rats whose mothers received a low-protein diet (1.90-fold increase in the LP group compared with the NP group). At PND 120, LP animals showed a slight decrease in this steroid hormone (0.87-fold reduction in the LP group compared with the NP group) (data not shown).

It was not possible to measure serum estradiol levels in 44-day-old animals because the values of this steroid hormone were below the lower limit of detection of the chemiluminescence technique (0.017 ng/dl). This occurred for both LP and NP animals, impeding comparison between the groups.

### 3.4 Impact of the Maternal Low-Protein Diet on AR, ERα and ERβ Immunolocalization

Immunolocalization of AR, ERα and ERβ was observed in the nuclei of epididymal epithelial cells and in epididymal interstitial cells at all analyzed ages. Furthermore, in the 44- and 120-day-old animals, these receptors were also labeled in peritubular smooth muscle cells.

#### 3.4.1 AR Immunolocalization

**FIGURE 4:**
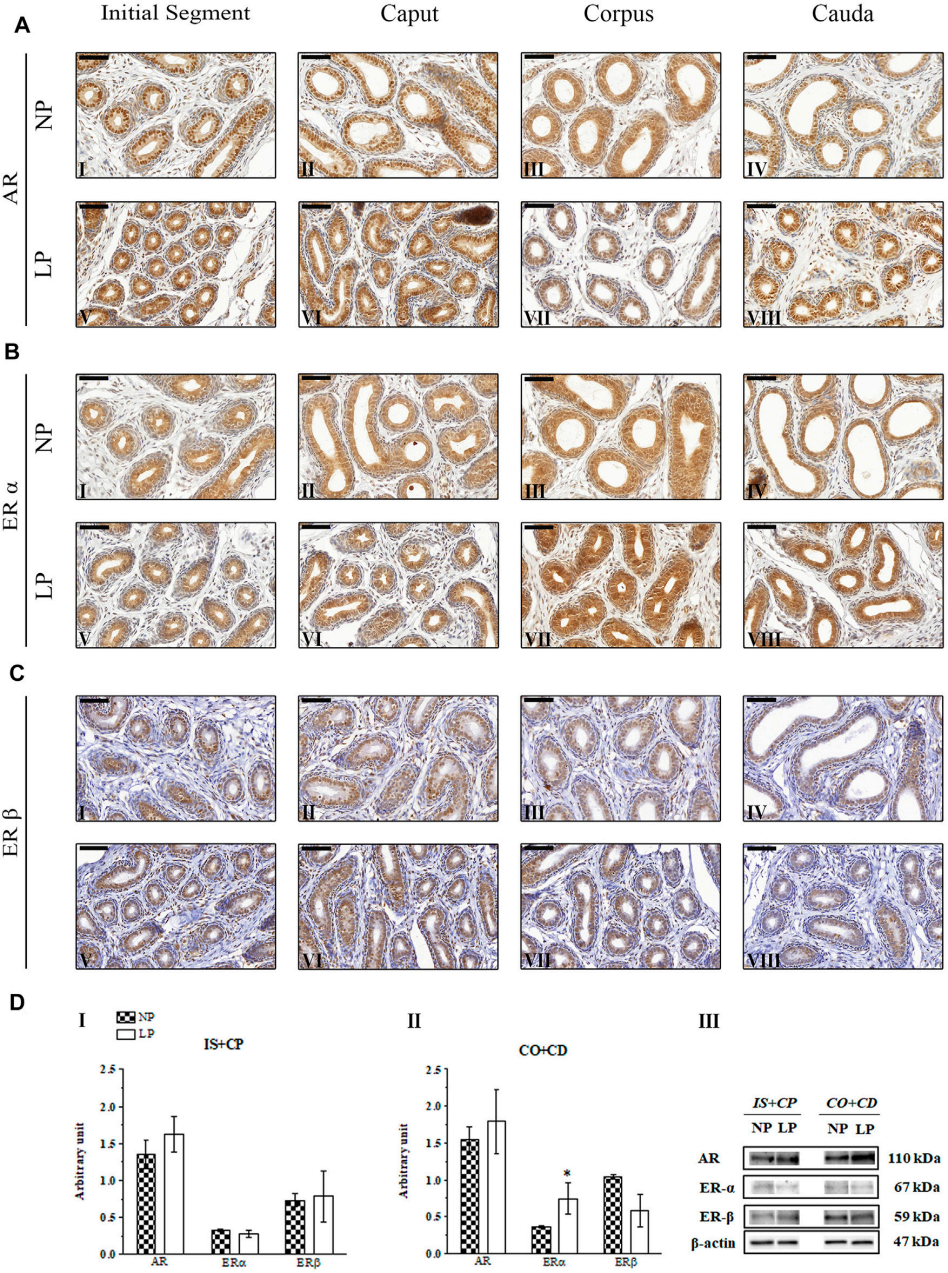
Expression and immunolocalization of AR, ERα and ERβ in the epididymides of 21-day-old animals. **(A)** Immunoreactivity for AR in epithelial cells and in mesenchymal cells in the initial segment (**I and V**), caput (**II and VI**), corpus (**III and VII**) and cauda (**IV and VIII**) regions of NP and LP animals. **(B)** Immunoreactivity for ERα in epithelial cells and in mesenchymal cells in the initial segment (**I and V**), caput (**II and VI**), corpus (**III and VII**) and cauda (**IV and VIII**) regions of NP and LP animals. **(C)** Immunoreactivity for ERβ in epithelial cells and in mesenchymal cells in the initial segment (**I and V**), caput **(II and VI**), corpus (**III and VII**) and cauda (**IV and VIII**) regions of NP and LP animals. Bar = 50 µm. **(D)** Extracts obtained from individual animals were used for densitometric analysis of the levels of the proteins in the initial segment plus caput (**I**) and the corpus plus cauda (**II**) regions following normalization to the levels of the housekeeping protein β-actin. The representative blots show the protein levels of AR, ERα, ERβ and β-actin (**III**, right panel). The data are presented as the mean ± S.E.M. *p < 0.05, Mann-Whitney test.

In both groups at PND 21, the AR-labeling pattern appeared more intense and uniform in epididymal epithelial cells, whereas in mesenchymal cells, it was less intense and more heterogeneous; these findings are consistent with those of previous studies investigating the immunolocalization of AR in the epididymides of young rats (You and Sar, 1998; Zaya et al., 2012). However, the AR-labeling intensity was slightly increased in the epididymal caput and cauda in LP animals of this age compared to NP animals of this age (Figure 4A LP VI and VIII; NP II and IV).

**FIGURE 5:**
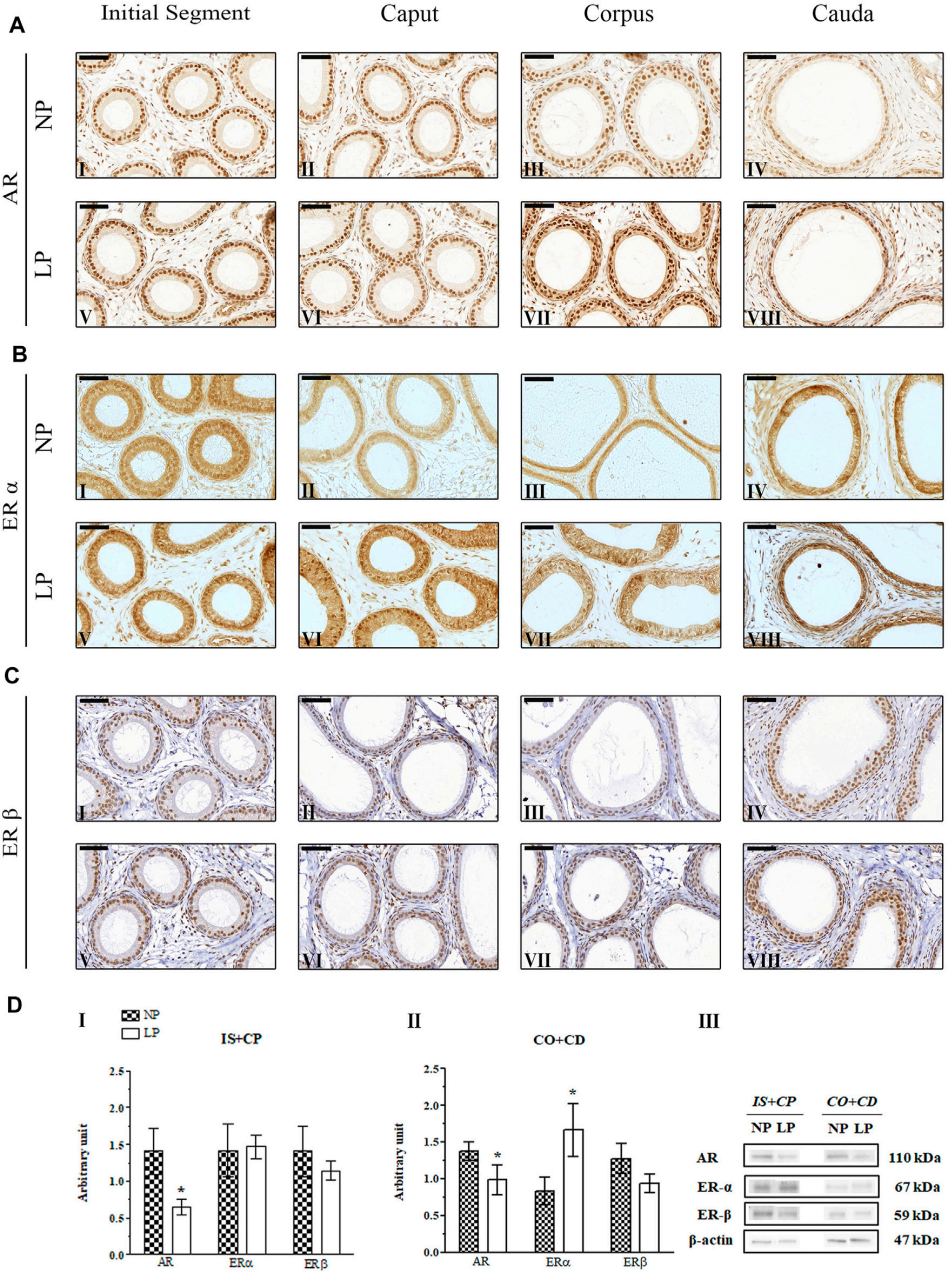
Expression and immunolocalization of AR, ERα and ERβ in the epididymides of 44-day-old animals**. (A)** Immunoreactivity for AR in epithelial cells and in mesenchymal cells in the initial segment **(I and V)**, caput **(II and VI)**, corpus **(III and VII)** and cauda **(IV and VIII)** regions of NP and LP animals. **(B)** Immunoreactivity for ERα in epithelial cells and in mesenchymal cells in the initial segment **(I and V)**, caput **(II and VI)**, corpus **(III and VII)** and cauda **(IV and VIII)** regions of NP and LP animals. **(C)** Immunoreactivity for ERβ in epithelial cells and in mesenchymal cells in the initial segment **(I and V)**, caput **(II and VI)**, corpus **(III and VII)** and cauda **(IV and VIII)** regions of NP and LP animals. Bar = 50 µm. **(D)** Extracts obtained from individual animals were used for densitometric analysis of the levels of the proteins in the initial segment plus caput **(I)** and corpus plus cauda **(II)** regions following normalization to the levels of the housekeeping protein β-actin. The representative blots show the protein levels of AR, ERα, ERβ and β-actin (**III**, right panel). The data are presented as the mean ± S.E.M. *p < 0.05, Mann-Whitney test.

In the 44-day-old NP animals, AR immunolocalization was observed in the nuclei of epididymal epithelial cells, with slightly more intense staining in the caput and cauda regions than in the other regions of the epididymis, corroborating the findings of Yamashita (2004), Perobelli et al. (2013) and Leite et al. (2014) (Figures 5AI,IV). Although we observed significant reductions in AR expression in the epididymal IS + CP and CO + CD regions of LP animals at PND 44 (Figures 5CI,II), there were no differences in the labeling pattern for this receptor between the NP and LP groups at this age (Figure 5A).

**FIGURE 6:**
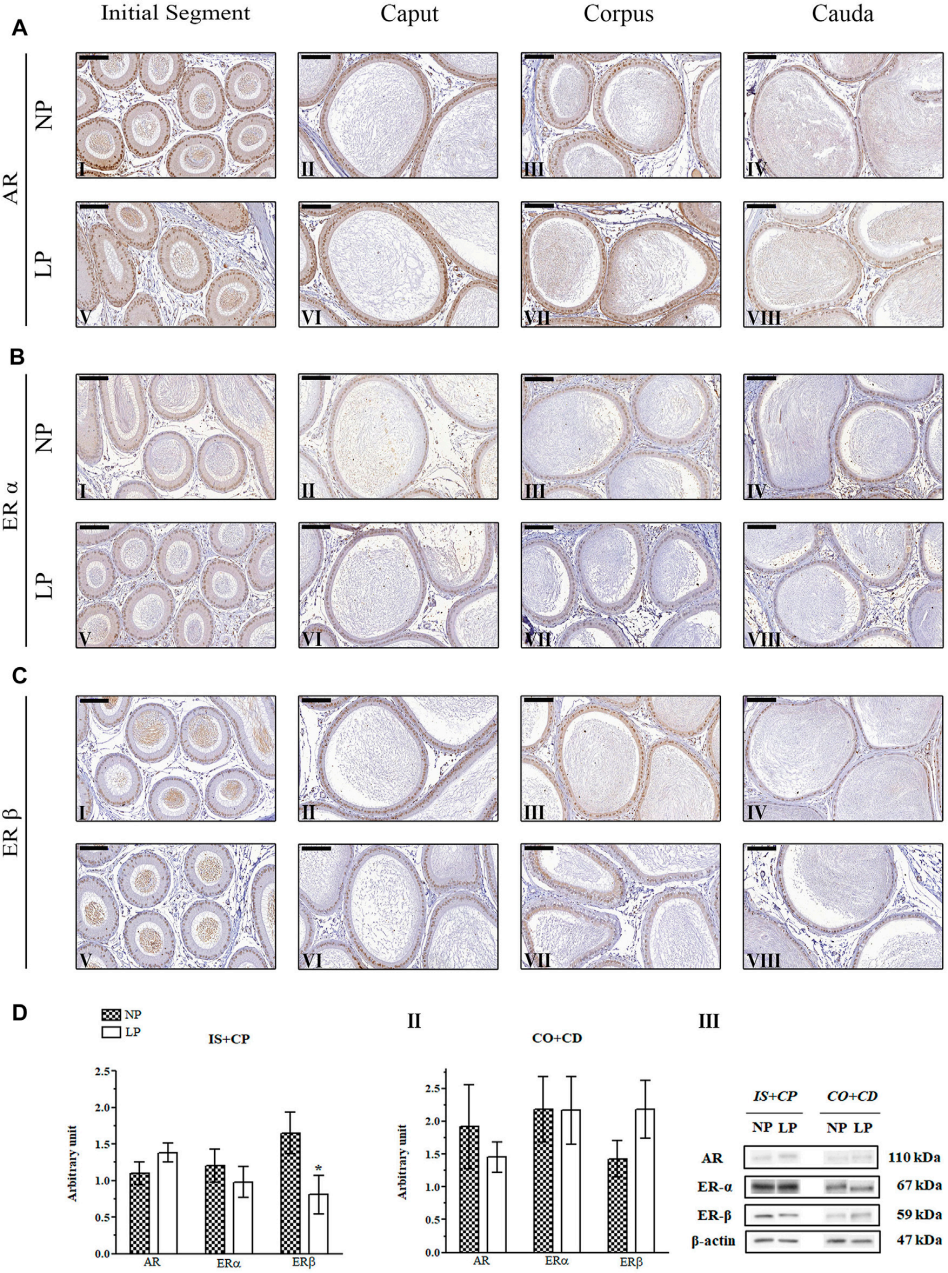
Expression and immunolocalization of AR, ERα and ERβ in the epididymides of 120-day-old animals. **(A)** Immunoreactivity for AR in epithelial cells and in mesenchymal cells in the initial segment **(I and V)**, caput **(II and VI)**, corpus **(III and VII)** and cauda **(IV and VIII)** regions of NP and LP animals. **(B)** Immunoreactivity for ERα in epithelial cells and in mesenchymal cells in the initial segment **(I and V)**, caput **(II and VI)**, corpus **(III and VII)** and cauda **(IV and VIII)** regions of NP and LP animals. **(C)** Immunoreactivity for ERβ in epithelial cells and in mesenchymal cells in the initial segment **(I and V)**, caput **(II and VI)**, corpus **(III and VII)** and cauda **(IV and VIII)** regions of NP and LP animals. Bar = 100 µm. **(D)** Extracts obtained from individual animals were used for densitometric analysis of the levels of the proteins in the initial segment plus caput **(I)** and corpus plus cauda **(II)** regions following normalization to the levels of the housekeeping protein β-actin. The representative blots show the protein levels of AR, ERα, ERβ and β-actin (**III**, right panel). The data are presented as the mean ± S.E.M. *p < 0.05, Mann-Whitney test.

In the epididymal epithelia of 120-day-old NP and LP animals, the nuclear staining of AR was more intense and homogeneous in the principal cells than in the other cells throughout the organ (Figures 6AI–VIII). Clear cells of the cauda region showed quite heterogeneous staining ranging from very discrete nuclear labeling to absent labeling in these cells (Paris et al., 1994; Zhu et al., 2000; Kaushik et al., 2010; Zaya et al., 2012) (Figures 6AIV,VIII).

#### 3.4.2 ERα Immunolocalization

At PNDs 21 and 44, NP animals showed mesenchymal cells with ERα labeling that was heterogeneous, moderate and only nuclear, while in the epithelial cells, the staining for this receptor appeared in a homogeneous way in the nucleus and cytoplasm throughout the epididymis. In differentiated clear cells, ERα labeling was only nuclear (Zaya et al., 2012) (Figures 4BI–IV; Figures 5BI–IV). The ERα immunolocalization pattern observed in 21- and 44-day-old LP rats did not differ from that observed for NP rats at these ages. However, the intensity of cytoplasmic labeling in the cauda region was more intense in LP animals than in NP animals of both ages, staining even the clear cell cytoplasm of this region (Figure 4BVIII; Figure 5BVIII).

No differences were observed in the ERα immunolocalization pattern between NP and LP animals at PND 120. In both groups, nuclear and cytoplasmic ERα labeling was observed in epididymal epithelial cells, mainly in principal cells, in all regions of the organ (Hess et al., 1997; Kolasa et al., 2003). Nuclear labeling for this receptor was also observed in interstitial and peritubular smooth muscle cells throughout all regions of the epididymis (Hess et al., 1997; Hess et al., 2011; Zaya et al., 2012) (Figures 6BI–VIII).

#### 3.4.3 ERβ Immunolocalization

The same pattern of ERβ immunolocalization was observed for NP and LP animals at PND 21. The intensity of nuclear and cytoplasmic ERβ labeling in epididymal epithelial cells varied considerably, and some of these cells showed a complete absence of immunoreactivity. A decreasing gradient was observed in the nuclear ERβ staining intensity from the initial segment and caput to the corpus and cauda of the organ. Immunostaining was also observed in the mesenchymal cells surrounding epididymal ducts (Sar and Welsch, 2000; Zaya et al., 2012) (Figures 4CI–VIII).

There was no difference in the pattern of ERβ labeling in LP animals compared to NP animals at PND 44. In both groups, epithelial cells, mainly principal cells, showed homogeneous nuclear immunostaining and discrete cytoplasmic labeling throughout the epididymis, with elevated nuclear staining intensity in the cauda region. In addition, heterogeneous nuclear labeling was observed in mesenchymal and peritubular smooth muscle cells in all regions of the organ (Figures 5CI–VIII).

The 120-day-old NP and LP animals showed the same pattern of ERβ immunostaining. Nuclear and cytoplasmic labeling of this hormone receptor was observed in epithelial cells throughout the epididymis. Principal cells presented homogeneous nuclear staining, while the other epididymal epithelial cells, peritubular smooth muscle cells and interstitial cells showed heterogeneous labeling (Choi et al., 2001; Kolasa et al., 2003; Yamashita, 2004; Zaya et al., 2012) (Figures 6I–VIII). Nuclear labeling of epithelial cells in the caput region was weak in LP animals compared to NP animals (Figures 6CIV,VIII).

### 3.5 Maternal Low-Protein Diet Changes AR, ERα and ERβ Expression in the Epididymides of Offspring in an Age-dependent Manner

A low-protein diet during gestation and lactation significantly decreased AR expression in the IS + CP (0.50-fold decrease in the LP group compared with the NP group) and CO + CD (0.72-fold decrease in the LP group compared with the NP group) at PND 44 (Figures 5DI,II). In 21-day-old animals, maternal protein restriction slightly increased AR expression in the IS + CP (1.20-fold increase in the LP group compared with the NP group) and CO + CD (1.17-fold increase in the LP group compared with the NP group) (Figures 4DI,II). In addition, at PND 120, this experimental model resulted in a nonsignificant reduction in the AR levels in the CO + CD region of the epididymis (0.76-fold decrease in the LP group compared with the NP group) (Figures 6DII).

The low-protein diet significantly increased ERα expression in the CO + CD region at PNDs 21 and 44 (PND 21: 2.10-fold decrease in the LP group compared with the NP group; PND 44: 2.01-fold decrease in the LP group compared with the NP group) (Figure 4DII; Figure 5DII). ERα expression was only slightly reduced in the IS + CP region of LP animals at PND 21 (0.85-fold decrease in the LP group compared with the NP group) and in the IS + CP and CO + CD regions of LP animals at PND 120 (IS + CP: 0.82-fold decrease in the LP group compared with the NP group; CO + CD: 0.99-fold decrease in the LP group compared with the NP group) (Figure 4D Figures 6DI,II).

There was no difference in ERβ expression between NP and LP animals at PNDs 21 and 44. Only in 120 day-old animals was a significant decrease in ERβ expression observed in the IS + CP region (0.50-fold decrease in the LP group compared with the NP group) (Figures 6DI).

### 3.6 Aromatase p450, but Not 5α-Reductase, Expression Is Altered by Maternal Protein Restriction

**FIGURE 7:**
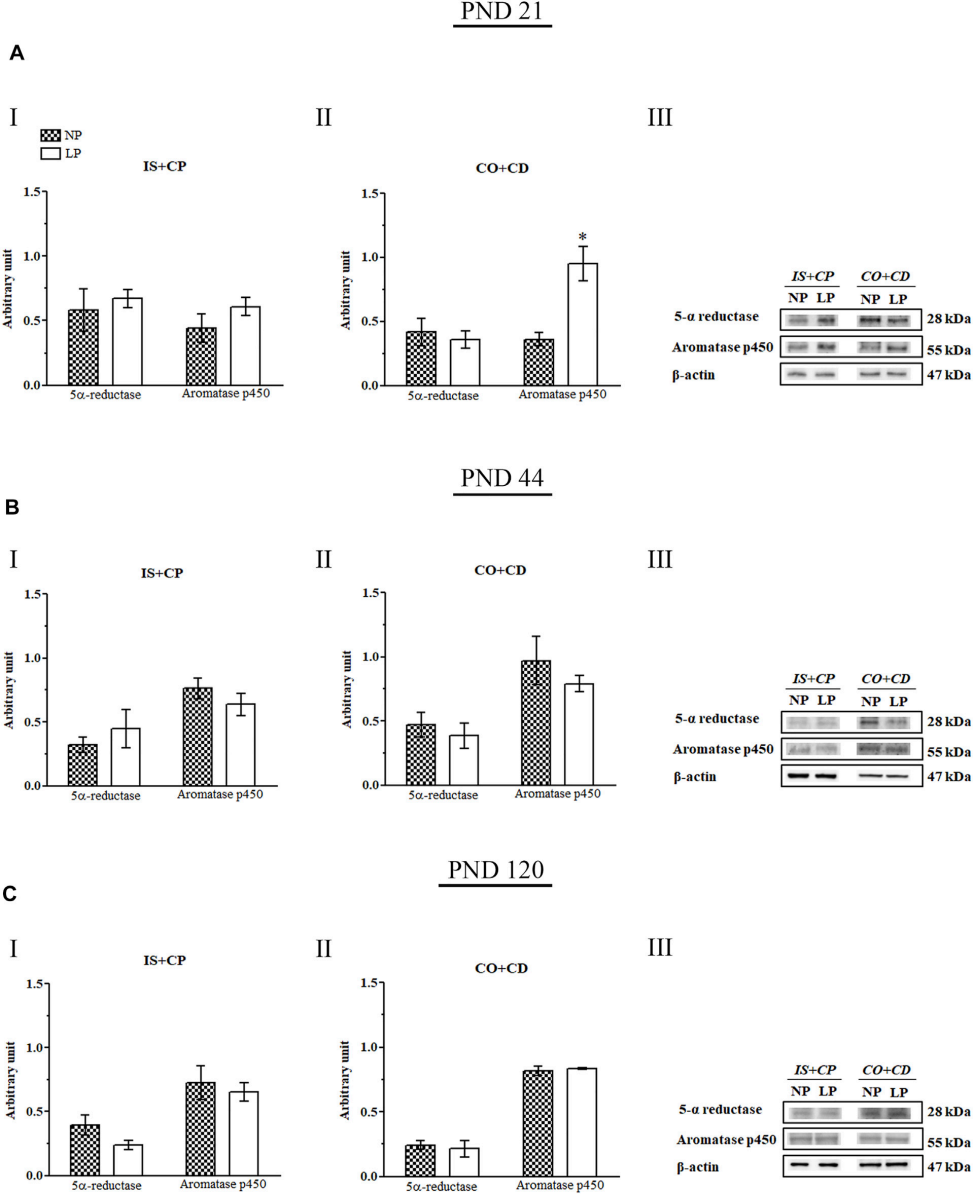
Immunoblots of 5α-reductase and aromatase p450. **(A)** Levels of 5α-reductase and aromatase p450 in the IS + CP **(I)** and CO + CD **(II)** epididymal regions of NP and LP animals on PND 21. The representative blots show the protein levels of 5α-reductase, aromatase p450, and β-actin (70 µg of protein) in 21-day-old animals **(III)**. **(B)** Levels of 5α-reductase and aromatase p450 in the IS + CP **(I)** and CO + CD **(II)** epididymal regions of NP and LP animals on PND 44. The representative blots show the protein levels of 5α-reductase, aromatase p450 and β-actin (70 µg of protein) in 44-day-old animals **(III)**. **(C)** Levels of 5α-reductase and aromatase p450 in the IS + CP **(I)** and CO + CD **(II)** epididymal regions of NP and LP animals on PND 120. The representative blots show the protein levels of 5α-reductase, aromatase p450 and β-actin (70 µg of protein) in 120-day-old animals **(III)**. The data are presented as the mean ± S.E.M. *p < 0.05, Mann-Whitney test.

A recently published study by our research group showed that maternal protein restriction during gestation and lactation significantly decreased serum testosterone levels in 44-day-old animals (Cavariani et al., 2019). However, the epididymal expression of 5α-reductase was unchanged at all of the analyzed ages (Figure 7). Notably, we observed a significant increase in aromatase p450 expression in the CO + CD region of the epididymis in PND 21 animals (2.64-fold increase in the LP group compared with the NP group) (Figures 7AII).

### 3.7 Maternal Protein Restriction Changed Both Src 416 and Src 527 Expression in an Age-dependent Manner

**FIGURE 8:**
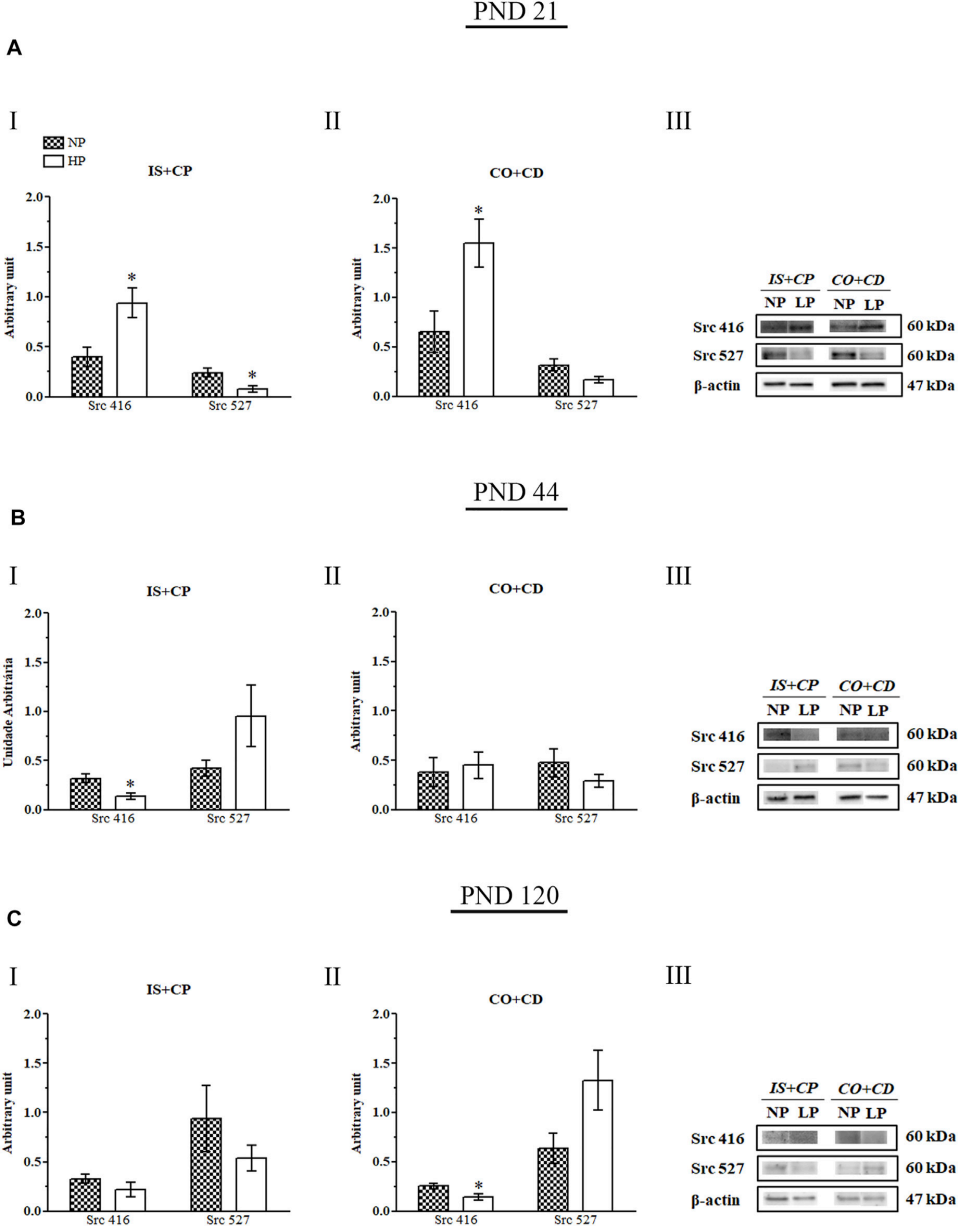
Immunoblots of Src 416 and Src 527. **(A)** Levels of Src 416 and Src 527 in the IS + CP **(I)** and CO + CD **(II)** epididymal regions of NP and LP animals on PND 21. The representative blots show the protein levels of Src 416, Src 527 and β-actin (70 µg of protein) in 21-day-old animals **(III)**. **(B)** Levels of Src 416 and Src 527 in the IS + CP **(I)** and CO + CD **(II)** epididymal regions of NP and LP animals on PND 44. The representative blots show the protein levels of Src 416, Src 527 and β-actin (70 µg of protein) in 44-day-old animals **(III)**. **(C)** Levels of Src 416 and Src 527 in the IS + CP **(I)** and CO + CD **(II)** epididymal regions of NP and LP animals on PND 120. The representative blots show the protein levels of Src 416, Src 527 and β-actin (70 µg of protein) in 120-day-old animals **(III)**. The data are presented as the mean ± S.E.M. *p < 0.05, Mann-Whitney test.

The low-protein diet increased Src 416 expression in the IS + CP (2.35-fold increase in the LP group compared with the NP group) and CO + CD (2.38-fold increase in the LP group compared with the NP group) regions of 21-day-old animals. In addition, at this same age, the Src 416 increase appeared to be accompanied by a decrease in Src 527 expression in the IS + CP region (0.33-fold decrease in the LP group compared with the NP group) in LP animals (Figures 8AI,II).

At PND 44, LP animals presented a significant decrease in Src 416 expression in the IS + CP region (0.44-fold decrease in the LP group compared with the NP group), while Src 527 expression was increased nonsignificantly in this region (2.26-fold increase in the LP group compared with the NP group) (Figure 8BII).

In adulthood (PND 120), we also observed a significant decrease in Src 416 expression accompanied by a nonsignificant increase in Src 527 expression in animals whose mothers received a low-protein diet during gestation and lactation (0.56-fold decrease in Src 416 levels in the LP group compared with the NP group; 2.06-fold increase in Src 527 levels in the LP group compared with the NP group). However, for PND 120, these results were observed in the CO + CD epididymal region of LP rats (Figures 8CII).

### 3.8. The Low-Protein Diet Increased Cldn-1 Expression Throughout the Epididymis

**FIGURE 9:**
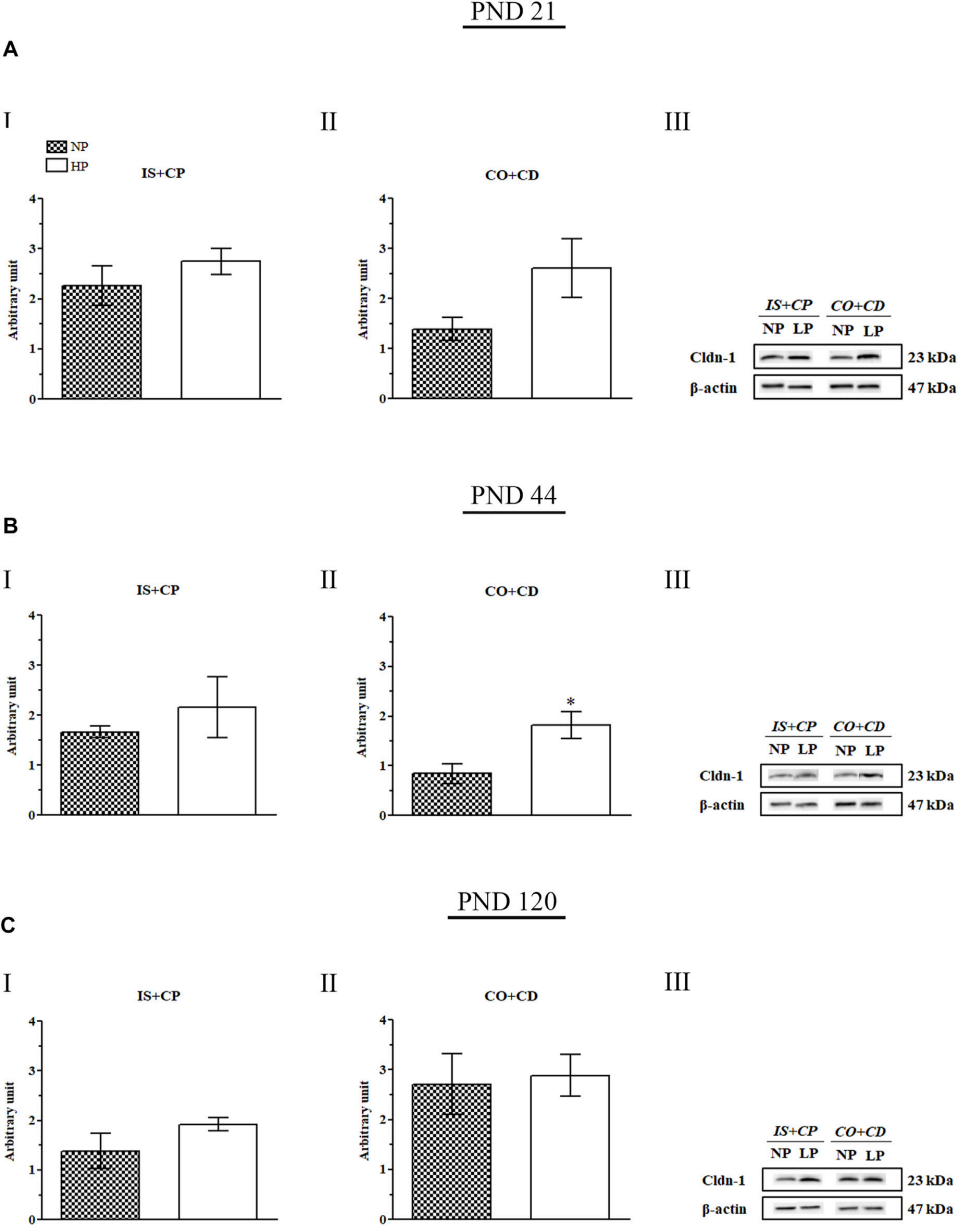
Cldn-1 immunoblot. **(A)** Levels of Cldn-1 in the IS + CP **(I)** and CO + CD **(II)** epididymal regions of NP and LP animals on PND 21. The representative blots show the protein levels of Cldn-1 and β-actin (70 µg of protein) in 21-day-old animals **(III)**. **(B)** Levels of Cldn-1 in the IS + CP **(I)** and CO + CD **(II)** epididymal regions of NP and LP animals on PND 44. The representative blots show the protein levels of Cldn-1 and β-actin (70 µg of protein) in 44-day-old animals **(III)**. **(C)** Levels of Cldn-1 in the IS + CP **(I)** and CO + CD **(II)** epididymal regions of NP and LP animals on PND 120. The representative blots show the protein levels of Cldn-1 and β-actin (70 µg of protein) in 120-day-old animals **(III)**. The data are presented as the mean ± S.E.M. *p < 0.05, Mann-Whitney test.

Maternal protein restriction during gestation and lactation increased Cldn-1 expression in the IS + CP and CO + CD epididymal regions at all ages analyzed. However, these results were only significant for Cldn-1 in the CO + CP region (2.17-fold increase in the LP group compared with the NP group) on PND 44. At this age, Cldn-1 expression was only slightly increased in the IS + CP region (1.3-fold decrease in the LP group compared with the NP group) (Figures 9BI,II).

Importantly, although the difference was not significant, Cldn-1 expression was increased in the IS + CP (1.22-fold increase in the LP group compared with the NP group) and CO + CD (1.88-fold increase in the LP group compared with the NP group) regions at PND 21 (Figures 9AI,II). This same pattern of Cldn-1 expression was observed in 120-day-old animals, both in the IS + CP (1.39-fold increase in the LP group compared with the NP group) and CO + CD (1.06-fold increase in the LP group compared with the NP group) regions (Figures 9CI,II).

## 4 Discussion

Low birth weight is an important sign of malnutrition during pregnancy and a crucial indicator of slow fetal growth (Jahan-Mihan et al., 2015). Adverse intrauterine nutritional conditions are able to program a series of adaptations in the developing fetus constituting an “economic” phenotype in order to increase the chances of immediate survival of the fetus and to grant advantages in a postnatal environment of nutritional scarcity (Hales and Barker, 1992). Therefore, the fetus interacts with the maternal environment dynamically in an attempt to predict the environment in which it is likely to be born, adapting for future competitive advantage (Gluckman and Hanson, 2006).

Maternal protein restriction is able to promote growth restriction at birth followed by the subsequent catch-up growth (Ozanne and Hales, 2004; Ozanne and Nicholas Hales, 2005). However, several studies with pregnant rats fed a low-protein diet reported pups with reduced body weight at birth which was maintained up to one year of ag (Zeman, 1967; Zambrano et al., 2005; Hoppe et al., 2007; Fetoui et al., 2009; Qasem et al., 2016; Yuasa et al., 2016). Consistent with these results, we observed lower body weight of the male offspring at birth after maternal protein restriction and at all analyzed ages. Our results showed that the components and quality of the maternal diet during critical periods of development may alter the development of offspring in the uterus and modify their phenotypes in adulthood.

Some studies have shown that maternal protein restriction does not alter offspring weight at birth, whereas others have demonstrated that insufficient protein intake during gestation and lactation significantly reduces the birth weights of both male and female pups (Burdge et al., 2003; Fernandez-Twinn et al., 2003; Torres et al., 2010; Whitaker et al., 2012; Rebelato et al., 2013; Claycombe et al., 2015; Gonzalez et al., 2016; Yuasa et al., 2016). The lower birth weight observed in the offspring whose mothers were fed a low-protein diet could be related to adaptation to a protein-deficient intrauterine environment, preparing the pups’ bodies to survive in a postnatal environment where the protein supply would also be low. Furthermore, during pregnancy, increased protein intake is recommended to attend the additional demand for nitrogen required by both mother and fetus (Jolly et al., 2004). With reduced protein intake, the pregnant rats of the LP group could not provide their developing offspring enough protein to reach a size similar to that of the offspring of rats fed a normal-protein diet. The CRLs of LP pups were also significantly lower than the CRLs of NP pups, consistent with their lower birth weights and with the results of other studies using this experimental model (Zeman, 1967; Colombelli et al., 2017).

AGD is a marker of sexual differentiation that reflects the action of androgenic hormones during the formation of the genital system in the uterus, being on average twice as large in males as in females (Graham and Gandelman, 1986; Welsh et al., 2008; Kita et al., 2016). Anogenital distance is usually regulated by testosterone produced by fetal testicles and is also affected by maternal androgens via the placenta (Graham and Gandelman, 1986). However, AGD reduction in LP pups could be correlated to the size of the pup independently of its intrauterine environment, since lighter and smaller animals tend to have significantly shorter AGDs than larger animals (Graham and Gandelman, 1986; Kerin et al., 2003; Dusek et al., 2010).

The difference in AGD and in CRL between NP and LP animals was maintained until PND 44, showing that maternal protein restriction during gestation and lactation delays the development of male offspring. However, when we observed the value of the AGD normalized by the CRL value, this difference no longer appear, indicating that at PNDs 21 and 44, the increase in AGD was proportional to the increase in CRL in LP animals, causing the ratio of these values to approach that of the values found for NP animals.

There is a maximum potential growth of anogenital distance programmed *in utero* (Scott et al., 2007; Kita et al., 2016). In male rats, AGD lengthens until PND 38, remaining constant from that period, and responds to hormonal stimuli during pubertal development, being negatively modulated by high doses of antiandrogens and testosterone (Kita et al., 2016). Thus, the maintenance of AGD reduction found in LP animals could be a consequence of protein restriction-induced programming during intrauterine life in addition to being a response to the slight increase in serum testosterone levels observed in 21-day-old animals. The decrease in AGD was maintained until PND 44, even with the significant reductions in testosterone levels observed in LP animals at this age [data related to testosterone levels are included in a recently published study from our research group Cavariani et al. (2019)].

Few epidemiological and experimental studies have addressed the effects of maternal protein restriction on offspring reproductive aspects, especially male reproductive aspects. Regarding the weight of genital system organs, the literature is controversial; studies have yielded results ranging from reductions in this parameter to no alterations in this parameter in animals whose mothers received a low-protein diet during gestation and lactation (Zambrano et al., 2005; Toledo et al., 2011; Rodriguez-Gonzalez et al., 2012; Rodriguez-Gonzalez et al., 2014).

The testes are the organs where gamete production occurs, while the epididymides are responsible for the storage, protection, concentration and maturation of these gametes. Therefore, the survival of mammals depends on these organs being fully functional (Cosentino and Cockett, 1986; Hermo and Robaire, 2002; Gatti et al., 2004). As previously mentioned, some effects of protein restriction are direct consequences of the alteration in the availability of substrate, and during pregnancy, increased protein intake is recommended to supply the requirements of rapid embryo growth (Godfrey and Barker, 2000; Jolly et al., 2004). In this context, the increased relative weight of these organs in 21-day-old LP animals could be an attempt to preserve their full functionality despite the poor nutritional environment to which the animals were exposed during their development and early postnatal life. However, even though the epididymal weight in relation to body weight was higher in LP animals at this age, the epididymal morphometry showed that the diameter of the epididymal duct, the diameter of the epididymal lumen, and the height of the epithelium were significantly smaller in LP animals compared to NP animals.

According to the “thrifty phenotype hypothesis”, adverse intrauterine nutritional conditions are able to program adaptations in the developing fetus to increase its chances of immediate survival and to confer advantages for its long-term survival in a postnatal environment of nutritional scarcity (Hales and Barker, 1992). The phenotype of an organism will tend to be normal if there is similarity between the pre- and postnatal environments. However, if the postnatal environment is incompatible with the predicted environment, fetal programming will make the organism susceptible to metabolic diseases (Armitage et al., 2005; Martin-Gronert and Ozanne, 2010; Qasem et al., 2012). Indeed, several studies have shown that a maternal low-protein diet during gestation and lactation can lead to permanent metabolic changes in offspring, even if the offspring have access to normal-protein diets after weaning (Zambrano et al., 2005; Zambrano et al., 2006; Colombelli et al., 2017; Santos et al., 2018; Cavariani et al., 2019). In the current study, animals from both groups were fed a normal-protein diet after weaning (PND 21). The increase in protein intake by the animals of the LP group seems to have been enough to increase the weight of these animals in relation to the weight of their organs and to increase epididymal diameter and epithelial height, since the increases in the relative weights of the testes, epididymides, and seminal glands and the decreases in epididymal morphometry observed for LP animals at PND 21 no longer appeared in these animals at PNDs 44 and 120. However, we cannot affirm that this restoration was enough to prevent changes in the functions of these organs.

At 44 days, the absolute and relative weights of the empty seminal glands were lower in LP animals than in NP animals. As these organs are very sensitive to testosterone, these decreases could be directly related to the lower concentrations of this hormone observed in these animals (Cavariani et al., 2019). Rats at PND 120 are sexually mature, and their seminal glands are full of a fluid that contributes to the coagulation of ejaculated semen (Mann, 1974). The increase in empty seminal gland weight in 120-day-old LP animals could have been due to a higher concentration of this fluid in animals of this group, independent of their seminal gland size.

AR is a member of the steroid receptor superfamily that plays a key role in the action of androgenic hormones (Chang et al., 1995). Increases in testosterone levels appear to be accompanied by increased expression of AR (Bentvelsen et al., 1995; Wang et al., 2016) while decreases in testosterone levels are followed by reductions in the expression of this hormone receptor (Zhu et al., 2000; Liu and Wang, 2005). Thus, the nonsignificant increases in the expression of AR in the epididymal IS + CP and CO + CD regions observed in 21-day-old LP animals could have been a consequence of a nonsignificant increase in serum testosterone levels in these animals (Cavariani et al., 2019). Similarly, the reductions in AR expression in both the IS + CP and the CO + CD regions of the LP animal epididymides at PND 44 could have been related to the decreases in the levels of this androgenic hormone observed in these animals (Cavariani et al., 2019).

In the 120-day-old LP animals, the nonsignificant decrease in serum testosterone levels (Cavariani et al., 2019) appeared to be accompanied by a nonsignificant decrease in AR expression in the CO + CD region, consistent with the results obtained for the LP animals at PND 44. However, in the epididymal IS + CP regions of these animals, the AR expression was slightly increased, suggesting possible nonandrogenic regulation of this receptor. Although several studies have demonstrated a positive effect of testosterone on AR expression, the mechanisms by which this regulation occurs are still not completely understood (Bentvelsen et al., 1995; Zhu et al., 2000; Ezer, 2002; Heinlein and Chang, 2002; Takayama, 2017). Furthermore, in addition to testosterone, AR can be transactivated by Src kinase through Tyr 543 phosphorylation, thereby triggering an extensive set of AR-dependent genes (Guo et al., 2006; Chattopadhyay et al., 2017). Moreover, prostate samples of men with castration-resistant prostate cancer present increased Src pathway activity in tumors with low AR activity, suggesting that Src activity probably has a strong negative correlation with AR activity (Mendiratta et al., 2009). The slight increase in AR expression observed in the IS + CP region in LP animals at PND 120 could therefore have been a response to the slight decreases in both Src 416 and Src 527 expression found in this epididymal region.

Several studies have shown the existence of crosstalk between Src and AR (Guo et al., 2006; Chattopadhyay et al., 2017; Szafran et al., 2017). Src is able to mediate AR phosphorylation, resulting in nuclear translocation and AR-responsive transcription (Guo et al., 2006; Chattopadhyay et al., 2017). Recently, an inverse AR and Src regulatory network has been supported in which AR may act on Src through microRNA (miR) expression modulation, thus regulating Src expression in a posttranscriptional way (Gu et al., 2014; Liu et al., 2015; Siu et al., 2016). AR transcribes and regulates miR-1 and miR-203, which in turn decrease Src expression; therefore, AR negatively regulates Src through miRs (Liu et al., 2015; Siu et al., 2016).

Due to the slight increase in AR expression in LP animals at PND 21, we expected to find decreased Src expression. However, this decrease was noticed only for Src 527, while Src 416 was significantly increased. Src activity is regulated by tyrosine phosphorylation at two different sites with opposite effects. Phosphorylation of Y416 in the activation loop of the kinase domain promotes enzyme activation, whereas phosphorylation of Y527 in the carboxy-terminal tail renders the enzyme less active (Xu et al., 1997). The increase in Src 416 expression in LP animals at PND 21 could have been an attempt to preserve epididymal development and functionality in the face of the poor nutritional conditions to which these animals were exposed during their early postnatal life. This increase in Src 416 expression could also have been related to the increased relative epididymis weight observed in these animals.

After weaning, both NP and LP animals were fed normal-protein diets. The increase in protein supply appeared to have been enough to meet the epididymal growth needs at PND 44 as well as the epididymal maintenance needs in adulthood, as we observed decreased Src 416 expression in the IS + CP region in 44-day-old LP animals and in the CO + CD region in 120-day-old LP animals. In addition, the decreased Src 416 appeared to be accompanied by a nonsignificant increase in Src 527 expression and by a decrease in AR expression in those same regions. Importantly, Src is incorporated into sperm during sperm maturation in the epididymides, being essential for sperm motility and for sperm function in fertilization (Krapf et al., 2012). Therefore, the decrease in Src 416 expression in the CO + CD regions of LP animals at PND 120 could have been related to sperm alterations previously observed by other authors using this experimental model (Toledo et al., 2011; Rodriguez-Gonzalez et al., 2014), which could compromise the fertility of adult animals whose mothers were subjected to protein restriction during gestation and lactation.

Although testosterone is the main regulating hormone of epididymal functions, estrogen is also produced and acts in males, regulating the functions of the epididymides, particularly those related to the reabsorption activity that occurs in these organs (Hess and Zhou, 2002; Cooke et al., 2017). In immature males, the main sources of estrogen are Sertoli cells, while in adults, germ cells show elevated expression of the enzyme aromatase, being the major sources of this steroid in the male genital system (Nitta et al., 1993; Hess et al., 1995; Janulis et al., 1998). Estrogen acts through ERα and ERβ nuclear receptors, both of which are present in the epididymides (Kuiper et al., 1996; Pelletier et al., 2000; Zaya et al., 2012).

Administration of estradiol to adult male rats results in increased ER expression in the genital systems of these animals (Cardone et al., 1998; Kaushik et al., 2010; Falvo et al., 2016). Similarly, antiestrogenic substances can reduce ER expression, demonstrating the ability of estrogens to regulate the expression levels of their own receptors (Zhang et al., 2013). In addition, androgenic regulation also has an impact on the expression of ERα and aromatase p450, with this type of receptor and this enzyme, respectively, being positively modulated by testosterone in organs of the male genital system (Shayu and Rao, 2006; Kaushik et al., 2012; Falvo et al., 2016). A recently published study by our research group showed that maternal protein restriction during gestation and lactation significantly decreased serum testosterone levels in 44-day-old animals (Cavariani et al., 2019).

Thus, the discrete increase in estradiol levels accompanied by the slight increase in serum testosterone levels in 21-day-old LP animals (Cavariani et al., 2019) could have been responsible not only for the increased expression of the aromatase p450 throughout the epididymides but also for the increase in ERα expression observed in the CO + CD epididymal regions of these animals.

Serum estradiol levels in the 44-day-old animals were below the detection level of the chemiluminescence technique and therefore could not be quantified. Although the lack of estradiol concentration at PND 44 impaired the effects of maternal protein restriction on these animals, the low levels were expected. In male rats, the period between PNDs 42 and 55 corresponds to the peripubertal period, and estradiol is very low, while testosterone reaches its peak (Dohler and Wuttke, 1975; Bell, 2018). However, a recent study published by our research group showed that, at this age, testosterone levels are significantly lower in animals whose mothers have received a low-protein diet than in animals whose mothers have received a normal-protein diet (Cavariani et al., 2019). The increase in ERα expression in the CO + CD region in LP animals at PND 44 could represent a compensatory mechanism given the reduced serum concentrations of testosterone. In adulthood, a protease removes DNA from the binding portion of ERα in the epididymides, showing that the action and influence of estrogen on these organs changes with age and that its action is greater during epididymal development than during adulthood (Hendry and Danzo, 1986; Schon et al., 2009). This mechanism may explain the fact that ERα expression was increased in the CO + CD regions of LP animals at PNDs 21 and 44 but did not remain altered at PND 120.

Regarding ERβ expression, no differences were observed between NP and LP animal epididymides at PNDs 21 and 44. Estrogens have the ability to differentially regulate the expression of the two ER types (Zhang et al., 2013). Thus, the decrease in ERβ expression observed in the IS + CP epididymal region in LP animals at PND 120 could have been a direct result of the slight decreases in serum estradiol levels observed in these animals without alteration of ERα expression.

Cldn-1 is a transmembrane protein that integrates the blood–epididymal barrier, whose structure and integrity are crucial for maintenance of the specific epididymal intraluminal environment (Hoffer and Hinton, 1984; Gregory and Cyr, 2006; Cyr et al., 2007; Dufresne and Cyr, 2007). It was observed that a low-protein diet increased Cldn-1 expression in the epididymides of the animals at all analyzed ages, although this increase was significant only in the CO + CD epididymal region at PND 44. Data regarding the expression patterns of AQPs 1 and 9 in a study using this same experimental model demonstrated that despite the fact that a decrease in AQP9 expression in the IS + CP region and increases in AQP1 and AQP9 expression in the CO + CD region were significant only for LP animals at PND 44, these changes were also observed in LP animals at 21 and 120 days of age. The decrease in AQP9 expression in the IS + CP region could have resulted in reduced water absorption; consequently, a greater amount of water could have been present in the epididymal lumen of this region and could have reached the epididymal CO + CD region, which could have led to the appearance of edema in the epididymides (Cavariani et al., 2019). Therefore, the increase in Cldn-1 expression observed in the epididymides of LP animals could have been an attempt to preserve the structure and conformation of the organ until AQP9 drained the excess water that was not absorbed by the IS + CP region, which would then be removed from the epididymal intertubular space by AQP1, preventing edema appearance and keeping the intraluminal environment balanced.

Collectively, the offspring of mothers who had limited protein intake during gestation and lactation showed reduced size and low birth weight remaining until adulthood. In addition, low genital organ weights were observed in rats at all ages, thus revealing the importance of maternal diet quality and showing that changes in the diet components can permanently affect the phenotype of an adult organism. Maternal protein restriction damaged the structure and functioning of the developing epididymis, since the expression of proteins associated with regulation, development and maintenance of the organ was altered in an age-dependent manner. Although some changes did not remain until adulthood, insufficient supply of proteins in early life impaired the structure and functioning of the epididymis in important periods of postnatal development, which may have contributed to the appearance of spermatic changes related to sperm motility, viability and concentration that could compromise the fertility of adult animals.

## Data Availability

The original contributions presented in the study are included in the article/Supplementary Material, further inquiries can be directed to the corresponding author.
