# Supplementary material for: Maternal Protein Restriction Alters the Expression of Proteins Related to the Structure and Functioning of the Rat Offspring Epididymis in an Age-Dependent Manner
Source: Front Cell Dev Biol. 2022 Apr 19;10:816637. doi: 10.3389/fcell.2022.816637 (PMC9061959; doi:10.3389/fcell.2022.816637)
Supplement: Supplementary file 1 [file Table1.DOCX]

Supplementary Material

| Crown-rump length and absolute and relative anogenital distance at PND 21 e 44. PND 21: NP, n = 17, LP, n = 22; PND 44: NP, n = 12, LP, n = 10. The values are expressed as the mean ± S.E.M. * p <0.05. T-testes were used to asses significance of differences in parametric data, and Mann-Whitney tests were used to asses significance of differences in nonparametric data. | | | | | | |
| --- | --- | --- | --- | --- | --- | --- |
| ***Parameters (mm)*** | | ***PND 21*** | | ***PND 44*** | | |
|  | | ***NP*** | ***LP*** | | ***NP*** | ***LP*** |
| Anogenital distance (mm) | 14.04 ± 0.43 | | 8.24 ± 0.36* | | 34.62 ± 1.07 | 25.58 ± 0.98* |
| Crown-rump length (mm) | 88.83 ± 1.56 | | 59.11 ± 1.54* | | 178.80 ± 0.37 | 142.50 ± 0.21* |
| Relative anogenital distance (mm) | 0.159 ± 0.006 | | 0.144 ± 0.006 | | 19.38 ± 0.050 | 18.04 ± 0.087 |
